# Valorization of Wild Edible Plants as Food Ingredients and Their Economic Value

**DOI:** 10.3390/foods12051012

**Published:** 2023-02-27

**Authors:** Jesús Clemente-Villalba, Francisco Burló, Francisca Hernández, Ángel A. Carbonell-Barrachina

**Affiliations:** 1 Research Group “Food Quality and Safety”, Centro de Investigación e Innovación Agroalimentaria y Agroambiental (CIAGRO-UMH), Miguel Hernández University, 03312 Orihuela, Spain; 2 Grupo de Investigación en Fruticultura y Técnicas de Producción, Centro de Investigación e Innovación Agroalimentaria y Agroambiental (CIAGRO-UMH), Miguel Hernández University, 03312 Orihuela, Spain

**Keywords:** antioxidants, edible flowers, economic value, minerals, principal component analysis, sugars, total phenol content, flavonoids

## Abstract

(1) Background: Wild Edible Plants (WEPs) are plants that grow without human help, by simply using the available resources. These types of plants are undervalued, because there is a lack of knowledge about their bioactive composition and nutritional/functional potential. (2) Scope and Approach: The main aim of this review is to fully identify the potential uses and importance of WEPs in certain regions based on (i) their sustainability, because they grow with their own resources, (ii) their content of bioactive compounds and consequently nutritional and functional value, (iii) their socio-economic relevance, and (iv) their ability to be useful in the agri-food industry in the short term. (3) Results: This review found evidence that a consumption of between 100 and 200 g of some of these WEPs can cover up to 50% of the recommended daily intake of proteins and fiber, being also a natural source of macro- and micro-minerals. Regarding their bioactive composition, most of these plants contain phenolic compounds and flavonoids, which determine their antioxidant capacity. (4) Conclusions: These reported results clearly demonstrate the high potential of the WEPs from a nutritional, economic and social point of view; although further studies are needed to gather deeper scientific information about their potential role in the socio-economic sustainability of specific groups of farmers worldwide.

## 1. Introduction

“Wild Edibles” is a term used to describe both plants and animals consumed by humans. In 1999, the Food and Agriculture Organization of the United Nations (FAO) described the term wild plants as “those that grow spontaneously in self-maintaining populations in natural or semi-natural ecosystems and can exist independently of direct human action” [1]. The FAO estimated in 2016 that as much as ~100 million people consumed wild edible plants (WEPs) in Europe [2]; this figure highlights the potential of these plants even currently. The consumption of this type of plants dates back to the Bronze Age as shown by the remains found in a site in Peñalosa (Jaén, Spain); more than 50 species were identified in this site, including *Rumex* sp. and *Calendula* sp. The conclusion of this ethnobotanical study was that these plants were used as food and/or as flavoring additives [3]. The importance of these plants is evident from multiple studies conducted worldwide, such as in Brazil [4], China [5], Ethiopia [6], Guatemala [7], Iceland [8], India [9], Japan [10], and Tunisia [11]. In Europe, Schulp et al. [12] wrote a review on the identification of WEPs throughout Europe, finding them in 17 countries. In fact, there are many studies focusing on the Mediterranean region because it has a great diversity of WEPs, especially in Greece, Italy, Portugal and Spain. In all of these studies, nutritional potential or bioactive profiles were reported [2,13,14,15,16,17]. Despite all the studies carried out over the last 10 years, the full potential of WEPs has still not been fully reached and this is a hot topic that deserves deeper attention by the scientific community considering especially the economy and their role in the sustainability of rural areas.

These plants may play an important role in environmental sustainability as they grow wildly and can be used as a functional ingredient to develop new food products. This sustainable character is persuading more and more consumers, chefs and nutritionists to introduce WEPs in their dishes and the diets they prescribe. The relevance of this review is supported by the growing global demand for a change in eating habits, where key new trends are essential and include: (i) reduction in gasses’ emissions, (ii) growth of sustainable crops, and (iii) greater environmental awareness. An example of this change and this new trend is the guide that FAO published in 2017 on wild food plants, or current campaigns for the consumption of edible insects as a new and sustainable source of protein [18]. The future must be more sustainable and WEPs can make a significant contribution to this change.

## 2. Scientific Literature Review

**Figure 1:**
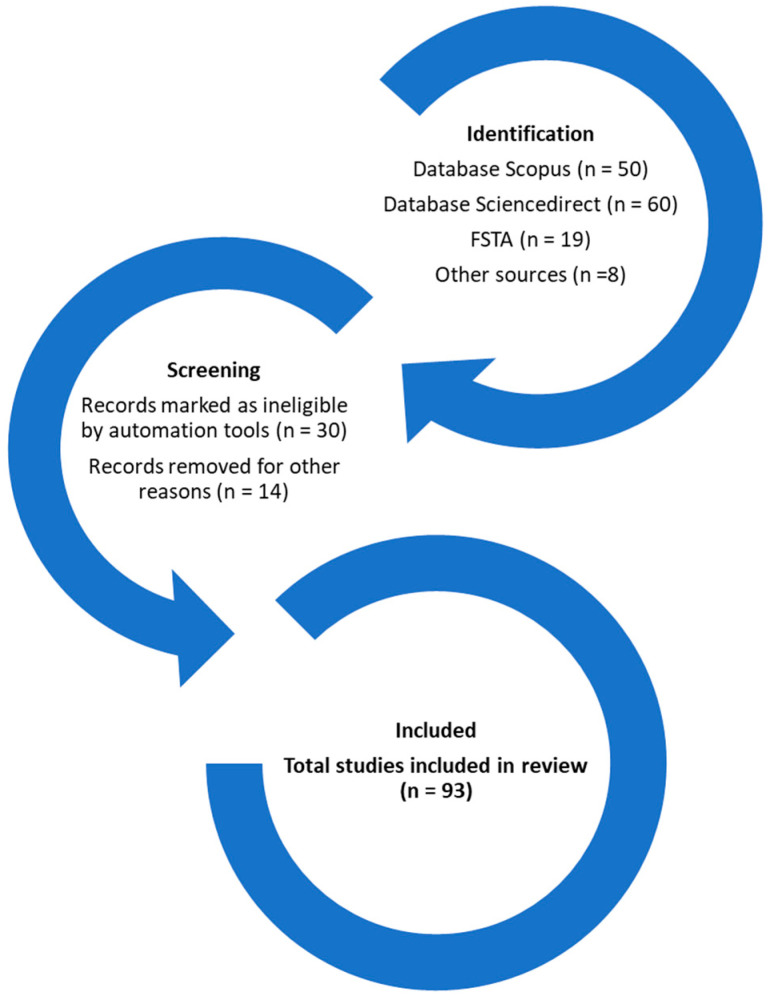
Diagram of selection of scientific publications.

To identify interesting scientific publications dealing with the composition and relevance of WEPs, this review was based on the 2020 update of the PRISMA approach [19]. The literature was searched in different databases: (i) Scopus, (ii) FSTA and (iii) ScienceDirect; the keywords used were the following: “wild edible plants”, “WEP”, “edible plants”, “ruderal plants”, and “wild edible plants food”. Most of the articles that were selected (1999 to 2022) were included in the Journal Citation Reports (JCR). The selection process is shown in Figure 1. The review is structured in different sections: (i) proximate characterization, (ii) sugars and organic acids, (iii) mineral content, (iv) fatty acids, (v) phenolic content and flavonoids, and (vi) economic value of WEPs.

## 3. Wild Edible Plants, WEPs

This review collects information on 115 WEPs (Table S1, Supplementary Material) located around the world and belonging to 47 different families. The families with the highest representation were Asteraceae > Brassicaceae > Fabaceae > Lamiaceae.

## 4. Proximate Characterization

Proximate analysis is used in foods to estimate the values of energy, moisture, protein, lipids, water, ash, and carbohydrate in the samples under study. Proteins, lipids and carbohydrates contribute to the total energy content in an organism, while ash and water also contribute to the organism mass [20]. Therefore, these determinations are essential for correct nutritional labelling, whose main objective is to provide data on macro- and micro-nutrients [21]. Nutritional labelling is regulated in the United States by the Food and Drugs Administration (FDA) and in the European Union by the European Food Safety Authority (EFSA). The main values reflected on food labels are: energy, protein, total fat, saturated fat, total carbohydrate, total sugars and sodium; all are expressed in g, mg or µg per 100 g [22].

The different parameters of the proximate characterization were compiled and summarized for 47 WEPs. The families with the highest representation in the proximate characterization were Asteraceae (9) > Lamiaceae (5) > Polygonaceae (4) (Table 1).

The water content or **moisture** is one of the most important parameters in a plant. For a plant to produce 1 kg of organic matter, it needs to absorb 500 kg of water, which is subsequently eliminated by different processes (e.g., transpiration or evaporation) [23]. In herbaceous plants, the moisture content usually reaches ~90% of the fresh weight, and only on rare occasions (such as intense water stress conditions) it is below 70%. The functions of the water content in the plant are essential, because it maintains cell turgor, facilitates the transport of solutes through the plant, participates in the reduction of CO_2_ through photosynthesis, and even in the cooling of the leaves during hot hours [24]. The average moisture content in the WEPs averaged ~80%. It should be noted that, out of the 115 WEP reviewed, the highest content was found in *Silybum marianum* (Asteraceae family) when it reached 93.4 g per 100 g [25,26]; on the contrary, the plant with the lowest moisture content was *Thymus pulegioides* (Lamiaceae family) with 47.6 g per 100 g [27]. In WEPs of the Lamiaceae family, it was observed that moisture values remained between 47.6 and 73.0 g per 100 g; these values were below the average (Table 1).

Studies dealing with 45 out of the 115 studied WEPs (from 24 families), provided results on ash contents. The two WEPs that showed the highest ash contents were: *Blumea lacera* (Asteraceae family) and *Hygrophila schulli* (Amaranthaceae family) with values of 24.05% and 23.36%, respectively [28]. On the contrary, the family that showed the lowest ash values was the Polygonaceae, which included four plants (*Rumex acetosella*, *Rumex induratus*, *Rumex papillaris*, and *Rumex pulcher*) with values of 1.2, 1.0, 1.0 and 1.9 g per 100 g, respectively [29,30].

**Table 1:** Proximate composition reported in wild edible plants, WEPs.

Plant Species	Part of Plant	Unit	Moisture	Ash	Proteins	Fat	Carbohydrates	Fibre	Energy ^§^	Reference
*Asystasia gangetica* (L.) T. Anderson	-	%	70.21 ± 0.98	17.35 ± 0.26	7.84 ± 0.12	2.04 ± 0.03	10.63 ± 0.23	8.14 ± 0.55	92.27 ± 0.27	[31]
*Achyranthes aspera* L.	-	%	53.34 ± 0.58	23.26 ± 0.65	12.60 ± 0.11	1.196 ± 0.01	14.35 ± 0.14	16.89 ± 0.34	118.62 ± 0.06	[31]
*Allium ampeloprasum* L.	Bulbs	g/100 g	78.3 (76.0–81.5)	0.8 (0.5–1.0)	1.7 (1.2–2.0)	0.18 (0.12–0.23)	16.6 (12.0–20.9)	4.2 (3.6–4.7)	-	[29]
*Allium ampeloprasum* L.	Bulbs	g/100 g	78.3 (76.3–80.3)	0.79 (0.59–0.99)	1.67 (1.31–2.03)	0.34 (0.13–0.61)	16.6 (12.8–19.7)	4.23 (3.72–4.74)	85 (65–103)	[17]
*Amaranthus viridis* L.	-	%	55.80 ± 0.23	13.31 ± 0.40	13.99 ± 0.12	1.40 ± 0.02	19.84 ± 0.07	6.54 ± 0.28	148.02 ± 0.28	[31]
*Anchusa azurea* Mill.	Leaves	g/100 g	91.2 (88.9–92.7)	1.9 (1.8–2.1)	1.9 (1.1–2.8)	0.15 (0.07–0.23)	1.3 (0.9–1.8)	3.9 (3.5–4.4)	-	[29]
*Apium nodiflorum* (L.) *Lag*	Stems	g/100 g	92.0 (90.0–94.0)	1.7 (1.0–3.3)	1.6 (1.1–2.1)	0.10 (0.07–0.14)	1.2 (0.7–2.1)	2.7 (1.9–3.4)	-	[29]
*Asparagus acutifolius* L.	Stems	g/100 g	84.6 ± 3.8	12.3 ± 0.0	22.4 ± 0.1	3.99 ± 0.33	61.3 ± 0.3	-	371 ± 1 ^‡^	[32]
*Asparagus acutifolius* L.	Shoots	g/100 g	85.4 (81.2–88.5)	2.23 (0.93–3.70)	2.40 (1.69–3.25)	0.61 (0.32–0.99)	3.56 (1.03–4.67)	4.83 (4.71–6.63)	40 (23–56)	[17]
*Beta maritima* L.	Leaves	g/100 g	87.3 (75.4–91.4)	2.68 (2.00–5.60)	3.10 (1.80–3.91)	0.34 (0.18–0.70)	1.71 (0.75–4.30)	4.38 (3.29–9.50)	31 (16–59)	[17]
*Beta vulgaris* subsp. *maritima*	Leaves	g/100 g	84.5 (75.4–89.1)	3.4 (2.0–5.6)	2.6 (1.8–3.6)	0.24 (0.16–0.40)	3.6 (2.9–4.3)	5.9 (3.9–9.5)	-	[29]
*Berberis aristata* DC.	Leaves	g/100 g dw ^ρ^	87.44 ± 2.22	15.46 ± 0.35	19.11 ± 0.78	2.14 ± 0.32	45.19 ± 0.56	18.10 ± 2.03	-	[28]
*Blumea lacera* (Burm. f.) DC.	Leaves	g/100 g dw	77.78 ± 2.68	24.05 ± 0.69	22.52 ± 0.97	0.93 ± 0.09	31.82 ± 1.26	20.68 ± 2.55	-	[28]
*Borago officinalis* L.	Leaves	g/100 g	86.9 (86.5–87.3)	2.4 (2.2–2.5)	1.2 (1.0–1.4)	0.16 (0.13–0.19)	9.5 (9.2–9.7)	-	44 ^‡^	[30]
*Borago officinalis* L.	Leaves	g/100 g	87.2 (86.4–88.8)	2.35 1.93–2.91	2.35 (1.93–2.91)	0.16 (0.13–0.19)	9.45 (7.23–10.7)	-	44 (34–50)	[17]
*Bryonia dioica* Jacq.	Stems	g/100 g dw	82.9 ± 2.3	8.79 ± 0.01	16.6 ± 0.4	15.1 ± 1.9	59.5 ± 1.2	-	440 ^‡^	[32]
*Bryonia dioica* Jacq.	Shoots	g/100 g	85.9 (70.9–90.8)	1.48 (1.00–3.30)	3.97 (1.00–11.9)	1.12 (0.10–2.90)	4.21 (0.80–10.37)	4.60 (3.40–10.7)	55 (14–141)	[17]
*Cichorium intybus* L.	Leaves	g/100 g fw ^γ^	86.4 (84.8–87.9)	1.8 (1.7–2.1)	2.9 (1.5–4.3)	0.13 (tr-0.25)	3.5 (1.8–4.7)	6.1 (5.1–6.7)	157 (137–180)	[25]
*Cichorium intybus* L.	Leaves	g/100 g	87.9 (75.0–94.5)	1.65 (1.25–2.10)	1.83 (0.20–4.30)	0.46 (Traces–0.92)	3.50 (1.80–4.7)	3.6 (1.20–6.70)	33 (10–58)	[17]
*Chondrilla juncea* L.	Leaves	g/100 g	87.8 ± 0.88	1.8 ± 0.11	1.9 ± 0.07	0.5 ± 0.04	2.0 ± 0.08	5.8 ± 0.32	19.6	[33]
*Chondrilla juncea* L.	Leaves	g/100 g	83.4 (65.9–89.7)	2.41 (1.39–4.35)	2.50 (1.83–6.13)	0.80 (0.09–1.50)	3.58 (1.49–9.69)	7.70 (4.10–13.4)	44 (22–104)	[17]
*Cynara cardunculus* L.	Flowers	g/100 g fw	84.94	1.13	3.27	0.15	10.51	5.4	47	[34]
*Enhydra fluctuans* Lour.	-	%	67.69 ± 0.78	15.15 ± 0.44	8.00 ± 0.06	1.10 ± 0.01	9.64 ± 0.06	15.37 ± 0.21	80.53 ± 0.16	[31]
*Erythrina variegata* L.	Leaves	g/100 g dw	87.44 ± 2.22	20.15 ± 0.53	21.12 ± 1.58	1.55 ± 0.15	39.63 ± 1.11	17.55 ± 1.98	-	[28]
*Foeniculum vulgare* Mill.	Leaves	g/100 g	86.7	-	3.8	-	4.9	3.5	48	[35]
*Foeniculum vulgare* Mill.	Leaves	g/100 g	82.4 (72.9–90.1)	2.34 (1.50–2.41)	2.76 (0.60–4.20)	0.42 (0.08–0.80)	9.67 (1.40–22.4)	3.87 (2.70–6.20)	63 (14–130)	[17]
*Glechoma hederacea* L.	Leaves	g/100 g	73.1 ± 8.05	3.47 ± 0.1	1.34 ± 0.00	1.18 ± 0.23	21.0 ± 0.17	-	99.96 ± 0.80	[27]
*Humulus lupulus* L.	Leaves	g/100 g	85.5 (85.2–93.2)	1.4 (0.9–2.0)	4.3 (3.1–5.1)	0.20 (0.11–0.26)	1.6 (1.4–1.8)	5.2 (4.3–6.4)	-	[29]
*Humulus lupus* L.	Shoots	g/100 g	85.8 (85.0–93.4)	1.35 (0.90–2.01)	4.25 (3.13–5.10)	0.37 (0.10–1.08)	1.85 (1.40–2.20)	4.85 (4.35–6.42)	39 (29–55)	[17]
*Hygrophilla schulli* (Hamilt.) M.R. Almeida & S.M. Almeida	Leaves	g/100 g dw	91.23 ± 1.01	23.36 ± 0.66	17.19 ± 1.49	1.92 ± 0.18	43.46 ± 0.42	14.07 ± 1.21	-	[28]
*Ipomoea aquatica* Forssk.	-	%	69.11 ± 0.72	16.37 ± 0.67	13.82 ± 0.08	2.19 ± 0.08	10.51 ± 0.08	7.44 ± 0.27	117.27 ± 0.24	[31]
*Malva sylvestris* L.	Flowers	g/100 g	72.49 ± 1.89	10.54 ± 0.30	8.50 ± 0.51	2.84 ± 0.37	78.12 ± 0.44	-	372.02 ± 2.13 ^‡^	[36]
*Malva sylvestris* L.	Leaves	g/100 g	81.0 (75.7–86.9)	3.21 (2.32–5.44)	3.00 (0.83–5.70)	0.56 (0.40–0.76)	2.23 (1.93–2.44)	4.76 (4.18–5.34)	35 (23–50)	[17]
*Mentha pulegium* L.	Inflorescences	g/100 g	59.47 ± 9.22	5.92 ± 0.09	7.12 ± 0.49	2.22 ± 0.22	84.74 ± 0.59	-	387.44 ± 0.53 ^‡^	[37]
*Montia fontana* subsp. *amporitana* Sennen	Leaves	g/100 g	91.47 ± 1.18	1.13 ± 0.13	1.76 ± 0.14	1.94 ± 0.13	1.81 ± 0.55	4.44 ± 0.34	31.48 ± 1.18	[38]
*Nasturtium officinale* R. Br.	Leaves	g/Kg	931 ± 10	9.4 ±0.9	22.4 ± 0.7	1.43 ± 0.08	35.6 ± 0.9	-	1023 ± 15 ^¤^	[39]
*Oldenlandia corymbose* Aiton.	-	%	60.28 ± 0.40	8.34 ± 0.39	10.52 ± 0.10	2.16 ± 0.06	9.08 ± 0.37	7.26 ± 0.30	97.94 ± 0.04	[31]
*Origanum vulgare* L.	Leaves	g/100 g	51.82 ± 5.11	2.87 ± 0.07	2.28 ± 0.03	2.81 ± 0.33	40.22 ± 0.28	-	195.31 ± 0.96	[27]
*Papaver rhoeas* L.	Leaves	g/100 g	91.0	-	2.9	-	3.1	2.5	36	[35]
*Papaver rhoeas* L.	Leaves	g/100 g	88.3 (68.5–91.2)	2.50 (1.45–5.20)	3.50 (1.50–5.90)	0.64 (0.15–1.03)	3.35 (2.90–5.30)	4.40 (2.50–11.10)	42 (24–78)	[17]
*Portulaca oleracea* L.	Leaves	%	81.5	28.9	27.8	0.141	-	10.0	-	[40]
*Portulaca oleracea* L.	Leaves	g/100 g	92.6 (90.0–94.3)	1.88 (1.25–2.95)	3.00 (2.50–3.50)	0.35 (0.30–0.40)	1.98 (1.11–2.70)	1.20 (0.90–1.80)	25 (19–32)	[17]
*Pterospartum tridentatum* (L.) Willk.	Flowers	g/100 g	60.8 ± 0.16	2.36 ± 0.00	15.92 ± 0.60	2.69 ± 0.51	79.03 ± 0.74	-	404.01 ± 4.05	[41]
*Rumex acetosella* L.	Leaves	g/100 g	89.9 ± 1.01	10.93 ± 1.06	7.85 ± 1.86	2.35 ± 0.28	78.87 ± 1.50	-	368.03 ± 3.98 ^‡^	[30]
*Rumex induratus* Boiss. & Reut	Leaves	g/100 g	90.29 ± 0.53	11.07 ± 0.30	13.54 ± 0.28	3.97 ± 0.14	71.42 ± 0.28	-	375.55 ± 0.36 ^‡^	[30]
*Rumex papillaris* Boiss. & Reut	Leaves	g/100 g	89.1 (87.8–90.7)	1.0 (0.4–1.3)	2.4 (1.6–3.5)	0.22 (0.26–0.28)	2.0 (1.6–2.7)	4.4 (4.0–5.0)	-	[29]
*Rumex pulcher* L.	Leaves	g/100 g	86.6 (87.4–89.2)	1.9 (1.1–3.1)	3.2 (1.9–5.5)	0.20 (0.10–0.32)	3.3 (1.5–4.5)	4.7 (4–5.2)	-	[29]
*Raphanus raphanistrum* L.	Leaves	g/100 g fw	89.9 ± 0.6	1.58 ± 0.08	4.04 ± 0.01	0.23 ± 0.03	4.22 ± 0.08	-	35.1 ± 0.1	[42]
*Scolymus hispanicus* L.	Leaves	g/100 g fw	84.1 (81.8–92.7)	3.19 (1.7–5.2)	1.8 (0.3–5.3)	0.09 (0.08–0.11)	3.4 (1.1–9.2)	7.0 (3.1–12.)	167 (53–280) ^¥^	[25]
*Sesbania sesban* (L.) Merr.	Leaves	g/100 g dw	90.13 ± 1.55	18.68 ± 0.22	15.65 ± 1.10	0.97 ± 0.05	49.51 ± 0.72	15.19 ± 1.79	-	[28]
*Silene vulgaris* (Moench) Garcke	Leaves	g/100 g	87.1 (80.4–88.5)	0.3	3.3 (3.0–3.6)	0.70	3.4 (2.9–3.9)	2.8 (2.6–3.1)	-	[43]
*Silene vulgaris* (Moench) Garcke	Leaves	g/100 g	85.9 (86.6–88.5)	1.53 (0.20–4.33)	2.47 (1.31–3.60)	0.67 (0.31–1.31)	2.32 (1.03–3.90)	4.36 (2.60–6.63)	34 (17–56)	[17]
*Silybum marianum* (L.) Gaertn.	Leaves	g/100 g fw	93.4 (92.9–93.8)	1.5 (1.0–1.9)	0.6 (0.5–0.8)	0.01 (tr-0.03)	1.1 (0.5–1.7)	2.6 (2.3–2.9)	51.8 (42.2–61.4) ^¥^	[25]
*Sonchus asper* L.	Leaves	g/Kg	864.3 ± 11.2	30.4 ± 3.0	32.5 ± 3.2	6.8 ± 0.7	19.8 ± 1.1	35.6 ± 2.2	1110 ± 120	[44]
*Sonchus oleraceus* L.	Leaves	g/Kg	872.4 ± 14.0	29.9 ± 1.8	31.7 ± 1.5	7.5 ± 0.9	18.2 ± 1.4	32.5 ± 2.4	1110 ± 120	[44]
*Sonchus oleraceus* L.	Leaves	g/100 g	87.6 (83.0–91.9)	2.17 (1.58–3.00)	2.22 (1.11–3.48)	0.60 (0.20–1.28)	2.29 (0.94–4.20)	3.37 (2.60–5.57)	33 (16–56)	[17]
*Sonchus oleraceus* L.	Leaves	g/100 g fw	88.25 (83.2–91.0)	2.04 (1.6–2.7)	2.22 (1.3–3.5)	0.29 (0.20–0.41)	2.51 (0.9–4.2)	4.3 (3.5–5.6)	127 (91–163) ^¥^	[25]
*Sonchus oleraceus* L.	Leaves	g/100 g wb ^ø^	89.3 ± 3.04	1.5 ± 0.01	3.0 ± 0.13	0.4 ± 0.01	-	5.5 ± 0.35	-	[45]
*Sonchus tenerrimus* L.	Leaves	g/Kg	877.3 ± 20.8	30.2 ± 1.9	31.8 ± 2.0	5.2 ± 0.4	13.2 ± 1.8	31. 2 ± 1.6	935 ± 105	[44]
*Tamus communis* L.	Leaves	g/100 g	86.2 (84.6–89.0)	1.4 (0.9–2.4)	3.2 (2.5–3.8)	0.17 (0.10–0.22)	2.2 (1.9–2.7)	4.7 (3.5–6.0)	-	[29]
*Tamus communis* L.	Shoots	g/100 g	85.2 (82.0–89.0)	1.25 (0.90–2.40)	3.13 (2.52–3.85)	0.49 (0.10–1.28)	5.20 (1.80–11.7)	4.35 (3.50–6.00)	46 (25–85)	[17]
*Taraxacum obovatum* (Willd.) DC.	Leaves	g/100 g fw	83.3 (79.2–86.7)	2.13 (1.8–2.5)	1.57 (1.02–2.09)	0.22 (0.19–0.27)	3.34 (1.63–5.39)	7.01 (5.4–8.7)	152 (114–205) ^¥^	[25]
*Thymus mastichina* L.	Leaves	g/100 g	54.67 ± 7.03	2.67 ± 0.08	2.2 ± 0.5	3.80 ± 0.10	36.64 ± 0.08	-	189.65 ± 0.44	[27]
*Thymus pulegioides* L.	Inflorescences	g/100 g	47.6 ± 12.60	4.94 ± 0.62	5.53 ± 1.40	0.18 ± 0.02	89.35 ± 1.54	-	381.14 ± 1.76 ^‡^	[37]
*Viola x Wittrockiana*	Flowers	g/100 g dw	87.76 (wet matter)	7.92	10.14	1.67	80.27	-	376.67 ^‡^	[4]
*Umbilicus rupestris* (Salisb.) Dandy	Leaves	g/100 g fw	93 ± 1	0.91 ± 0.01	1.83 ± 0.06	0.255 ± 0.002	3.90 ± 0.03	-	25.2 ± 0.1	[46]

^§^ kcal/100 g; ^‡^ kcal/100 g dw; ^¤^ kJ/kg; ^¥^ kJ/100 g; Mean value (minimum-maximum); ^ρ^ dw = dry weight; ^γ^ fw = fresh weight; ^ø^ wb = wet bases.

With respect to protein, three WEPs stood out for their high protein content, each belonging to a different family (Portulacaceae, Asteraceae, and Asparagaceae): *Portulaca oleracea* with 27.8 g per 100 g [40], followed by *Blumea lacera* and *Asparagus acutifolius* with 22.52 and 22.40 g per 100 g, respectively [28,32]. On the contrary, the plant with the lowest protein content was *Silybum marianum* (Asteraceae family), with a protein content as low as 0.6 g per 100 g [25,26].

Fat content was only reported in 47 of the available 115 WEPs. *Bryonia dioica* (Cucurbitaceae family) showed the highest content at 15.1 per 100 g dry weight (Martins et al., 2011). The family with the highest number of plants (Asteraceae) had, in general, low contents, reaching its maximum with *Silybum marianum* and *Enhydra fluctuans* (1.1 g per 100 g), and its minimum with *Scolymus hispanicus* (0.09 g per 100 g) [25,26,31].

The scientific literature only provides the carbohydrates content for 46 WEPs out of the 115 reviewed plants. In this regard, *Thymus pulegioides* and *Mentha pulegium* had the highest content, reaching 89.35 and 84.74 per 100 g, respectively [37]; both plants belong to the Lamiaceae family. Within this family, plants showed a wide variability in carbohydrate content, with *Thymus pulegioides* having the highest value (89.35 g per 100 g), while *Glechoma hederacea* had the lowest with 21 g per 100 g [27].

The content of dietary fiber was only found for 36 WEPs. The WEPs that stood out for their high fiber content were *Blumea lacera* (20.68 g per 100 g dw) and *Berberis aristata* (18.10 g per 100 g dw), belonging to the Asteraceae and Berberaceae families, respectively [28]. In general, the fiber content of the Laminaceae and Brassicaceae plants was not analyzed and thus was not reported [27,37,39,42].7

**Energy** is calculated from the determination of food macro-nutrients including protein, fiber, carbohydrates, fat and alcohol [47]. Nowadays, part of society is willing to have a balanced diet, but unfortunately, most consumers, due to their lifestyle, replace traditional diets with diets high in sugars and refined fats, which leads to large caloric intakes; these diets result in increased incidence of coronary heart disease, strokes, type II diabetes, and obesity [48,49]. However, in the USA the actual daily intake for men is 2800 kcal per day and for women 2000–2200 kcal per day, which are comparable to the 2030 kcal that a person 2 m tall and 88 kilos should ingest, this value being the highest of those established for men and women by the FAO [50]. This difference in daily kilocalories shows a gap between the ideal intake and that which is averaged in countries such as the USA. Energy data was only available for 28 WEPs out of the 115 plants reviewed. The plant with the highest energy value was *Bryonia dioica* with 440 kcal per 100 g dw [32]. Regarding families, the Polygonaceae showed high contents, for instance, *Rumex acetosella* and *Rumex induratus* with 368 and 376 kcal per 100 g dw, respectively [30].

## 5. Sugars and Organic Acids

### 5.1. Sugars

The role of the sugars, which are generated through the photosynthesis process, in plants is fundamental; they are the main source of carbon and energy for the plants, and participate in the plant metabolism control, for example participating in multiple biological processes, from embryogenesis to plant senescence [51,52]. Sugars can be classified into monosaccharides, disaccharides, and polysaccharides. Apart from the biological importance of sugars in plants, it is necessary to highlight their importance in the health of humans. Sugars are the main source of energy for multiple metabolic processes, as well as making a necessary contribution for cells to stay alive [53].

Sugar contents of 22 WEPs (from 16 families) are summarized in Table 2. In general, the predominant sugars were fructose, glucose and sucrose. It should be noted that, of the three main sugars, glucose was that with the highest total content in all 22 plants, followed by fructose and subsequently sucrose.

The most commonly studied plant family regarding sugars was Lamiaceae with five plants. Regarding fructose, the WEPs that had the highest content were *Malva sylvestris* (Malvaceae family) and *Tamus communis* (Dioscoraceae family), with 8.72 and 3.83 g/100 g dw, respectively [32,36]. *Malva sylvestris* also showed the highest glucose content at 7.35 g/100 g dw [36]. The sucrose concentration in these 22 WEPs was mainly dominated by two plants: *Mentha pulegium*, which had 4.62 g/100 g dw, and *Asparagus acutifolius* with a sucrose concentration of 4.27 g/100 g dw [32,37].

### 5.2. Organic Acids

At a general level, organic acids are weak acids, which can be classified mainly by four criteria: (i) nature of the carbon chain (aliphatic, aromatic, etc.); (ii) saturation or unsaturation properties; (iii) substituted or unsubstituted characteristics; and (iv) number of functional groups. Acids play an essential role in the physiology of plants, participating in processes such as pH regulation, balancing the redox potential cells, the Krebs cycle, or even in organoleptic properties such as color, taste and aroma of both fruits and vegetables [58,59,60].

Three organic acids (oxalic, malic and shikimic) were mainly found in WEPs, and especially in four families: Asteraceae, Brassicaceae, Crassulaceae, and Portulacaceae (Table S2).

**Figure 2:**
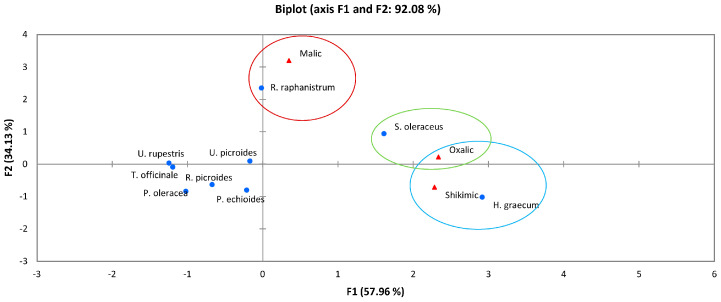
Principal Component Analysis (PCA) of organic acids present in wild edible plants, WEPs.

One of the most representative organic acids in the Asteraceae family is oxalic acid [25,26], which show a high content in six plants of this family [61]. Being a small group of plants in which organic acids were determined, a PCA (Principal Component Analysis) was carried out to understand these results in a visual way (Figure 2). The values can be found in Table S2. PCA showed a high percentage of correlation (92.08%) among organic acids and WEPs, being able to differentiate three groups. In the first cluster, malic acid was associated with *Raphanus raphanistrum* (Brassicaceae family); the second cluster was based on the association of oxalic acid with *Sonchus oleraceus* (Asteraceae family); while in the third group both oxalic acid and shikimic acid were associated with *Hymenonema graecum* (Asteraceae family).

In this way, the highest contents of malic acid were reported in *Raphanus raphanistrum* and *Sonchus oleraceus* (580 and 415 mg/100 g, respectively) [42,61]. The WEPs in which the highest amounts of oxalic acid were found, in decreasing order were: *Hymenonema graecum* > *Sonchus oleraceus* > *Raphanus raphanistrum*, with concentrations between 972 and 706 mg/100 g [42,61]. Regarding shikimic acid, two plants stood out for their contents: *Hymenonema graecum* and *Sonchus oleraceus*, with 244 and 166 mg/100 g, respectively; the other WEPs where this acid was found had values of below 100 mg/100 g [61].

## 6. Mineral Elements

The Mediterranean diet is considered by many experts as one of the best in the world at a nutritional level, and one of its main strengths is the contribution of minerals and vitamins [62]. In body composition, minerals represent fourth place in abundance, reaching values of up to 6.1% of the body weight for a person of 65 kg [50]. This importance of minerals for the human body determines health problems due to mineral deficiency; in this way, key elements which are often linked to deficiency are iron, zinc, and iodine. Iron deficiency can affect as many as 18% of the world’s children under 5 years of age, along with pregnant women [63]. In addition, zinc and iodine affect 17% and 28% of the world’s population, respectively [63]. According to Zeece [64], the main source of minerals is the soil, because from there it passes to plants and through the food chain to humans. The daily intake of minerals establishes the recommended intake for each of these elements; in Table S3, it is possible to find these values according to the Food & Drug Administration (FDA) [65].

**Figure 3:**
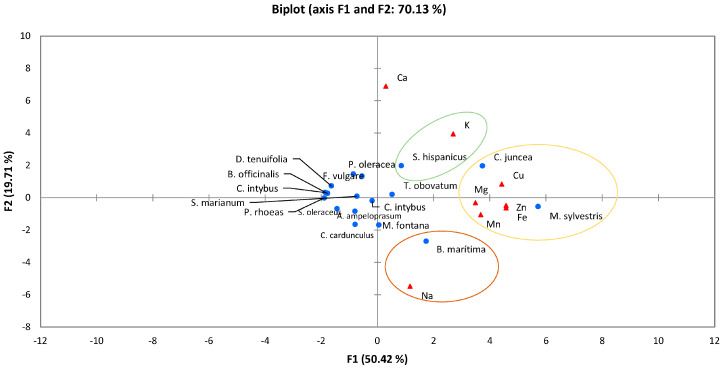
Principal Component Analysis of minerals present in wild edible plants, WEPs.

Principal Component Analysis (PCA) (Figure 3) was carried out after adjusting all values in a common unit, mg/100 g fw. PCA showed a total percentage of correlation (70.13%) among minerals contents and WEPs. Mineral composition was reported only for 17 of the 115 plants (from 10 families) covered in this review. In this way, most of the plants which had their mineral profile analyzed (n = 7) belonged to the Asteraceae family. The macro-elements identified were calcium (Ca), magnesium (Mg), potassium (K), and sodium (Na); while the micro-elements were copper (Cu), iron (Fe), manganese (Mn), and zinc (Zn). In Table S4 [25,34,35,38,57,66,67,68], values for the contents of these minerals can be found.

Regarding Figure 3, three different groups can be easily observed. On the one hand, Ca was isolated from the entire group of minerals as not being associated with any plant. The first group contained the highest number of minerals (Cu, Fe, Mn, Mn, and Zn). The second and third groups were linked to K and Na, respectively.

Calcium was not associated with any specific WEPs, although *Foeniculum vulgare* was that with the highest content of this mineral, followed by *Chondrilla juncea* (341 and 301 mg/100 g, respectively) [25,35].

The first group was mainly associated with two plants: *Chondrilla juncea* and *Malva sylvestris*. Regarding Cu and Mn, the WEPs that had the highest simultaneous contents, were *Chondrilla juncea* (0.43 and 0.97 mg/100 g, respectively) followed by *Malva sylvestris* (0.33 and 0.76 mg/100 g, respectively); although the WEP that had the highest Mn content was *Montia fontana* with 1.08 mg/100 g [38]. Regarding Fe, Mg, and Zn, *Malva sylvestris* had the highest contents (5.82, 715 and 1.98 mg/100 g, respectively) compared to *Chondrilla juncea* (3.97, 40.80 and 1.63 mg/100 g, respectively) [25]. In the second group, K was exclusively associated with *Scolymus hispanicus*, the content of which was much higher than in the rest of the WEPs (1040 mg/100 g) [25,26]. In the third group, Na was exclusively associated with *Beta maritima*, containing the highest concentration of this mineral, 171 mg/100 g) [66].

The mineral values clearly demonstrated that many WEPs have a high nutritional value, for example, *Chondrilla juncea*, which can provide almost 50% of the recommended daily intake of Cu after consumption of 200 g of the plant. *Montia fontana*, with its Mn content, could also cover approximately 50% of the recommended daily amount after consumption of 200 g (Table S3) [65].

## 7. Fatty Acids

Of the 115 WEPs evaluated in this review, only 43 (from 21 families) had FA profiles published; the most commonly studied families regarding FAs were: Asteraceae (8) > Lamiaceae (5) > Polygonaceae (4) > Brassicaceae (3). As can be seen in Table 3, the most representative FAs in WEPs were palmitic, oleic, linoleic, and linolenic acids.

Linolenic acid was by far the FA with the highest percentage in most plants, showing in eight of these 42 plants a percentage above 60%; the list of plants in decreasing order of abundance was as follows: *Bryonia dioica* Jacq. (70.3%) > *Malva sylvestris* (67.8%) > *Papaver rhoeas* (65.0%) > *Anchusa azurea* (64.7%) > *Rumex pulcher* (63.0%) > *Origanum vulgare* (62.3%) > *Umbilicus rupestris* (62.0%) > *Cichorium intybus* (62.0%) [27,32,46,56,70]. It is important to highlight that this compound was present in a wide range of families.

Linoleic acid was the second most abundant FA, highlighting its importance in WEPs, especially in *Allium ampeloprasum* (53.5%) > *Asparagus acutifolius* (44.5%) > *Tamus communis* (42.0%) [32,67,72]; these plants belong to the Amaryllidaceae, Asparagaceae and Dioscoreaceae families, respectively.

Another of the most representative FAs, palmitic acid, was present in all the WEPs analyzed. The WEP that obtained the highest value in palmitic acid was *Viola x wittrockiana* (Violaceae family) with 36.41 g/100 g dw [4]. *Silybum marianum* (Asteraceae), *Allium ampeloprasum* (Amaryllidaceae) and *Portulaca oleracea* (Portulaceae) had values of 28.7%, 26.4% and 24.7%, respectively [57,67,70,72].

Regarding oleic acid, the highest content was found in *Glechoma hederacea* with 35.1% [27], followed by *Portulaca oleracea* with 12% [57]; whilst, the WEP with the lowest content of oleic acid was *Umbilicus rupestris* (0.6%) [46].

In general, the main FAs identified in WEPs were linolenic and linoleic acids. On the other hand, it should be noted that palmitic acid was identified in all plants, although at lower concentrations.

## 8. Phenolic Content and Flavonoids

Phenolic compounds are the most abundant secondary metabolites in plants, helping in various functions of great importance, such as pigmentation, growth, and/or resistance to pathogens, also playing a fundamental role in maintaining redox homeostasis of cells [73,74,75]. Apart from the importance in the physiology of plants themselves, it has been clearly demonstrated that these compounds have antioxidant activity, anti-inflammatory effects, help in the reduction of oxidative stress, and can even help in the prevention of tumors [76,77,78,79].

In the references evaluated in this review, instudying up to 115 WEPs (from 47 families), the total phenolic contents (TPC) were provided for 100 plants; for 51 plants, the total flavonoids content (TFC) was also provided (Table 4) [5,30,32,36,39,42,46,54,56,57,67,69,72,80,81]. Regarding TPC and TFC, the most commonly studied families were Asteraceae (17) > Brassicaceae (7) > Fabaceae (6) = Lamiaceae (6). These analyses were performed by HPLC [31,54,81,82,83,84] and spectrophotometric method [4,5,28,30,32,36,37,39,41,56,57,67,69,72,80].

The WEP with the highest TPC was *Tamus communis* reaching 404 mg GAE/g, this plant being the only from the Diocoreaceae family [82]. Regarding the Asteraceae family (17 WEPs), the TPC ranged between 1.1 mg GAE/g (*Youngia japonica*) to 184 mg GAE/g (*Helichrysum stoechas*) [81,82]. It is necessary to highlight that the Polygonaceae family, although only data for four plants was available, showed values above the average, ranging between 73.44 mg GAE/g (*Rumex pulcher*) and 142 mg GAE/g (*Rumex acetosella*) [30,72].

Data regarding TFC (mg EC/g) were found for 31 WEPs, with *Origanum vulgare* (Laminaceae family) and *Malva sylvestris* (Malvaceae family) having the highest values at 224 and 211 mg CE/g, respectively [36,80]. Regarding the 7 WEPs whose flavonoids content was expressed in mg RE/g, the highest flavonoids content was shown by *Sophora viciifolia* (Fabaceae family) with values of 237 mg rutin/g dry extract [5]. On the other hand, of the 6 WEPs whose flavonoids content was expressed in mg of quercetin, three stood out for their high content: *Sesbania sesban* (Fabaceae family) > *Hygrophila schulli* (Acanthaceae family) > *Berberis aristata* (Berberidaceae family) (97.16, 87.12 and 82.05 mg QE/g, respectively) [28]. *Diplotaxis erucoides* (Brassicaceae family) and *Asparagus acutifolius* (Asparagaceae family) showed the highest content of flavonoids expressed in mg QE/kg WW, at 2877 and 2263, respectively [83].

These data show the great importance of the WEPs due to their bioactive composition, and also their great diversity, with so many families represented.

## 9. Economic Value of Wild Edible Plants (WEPs)

Nowadays, a fundamental aspect in society is the economy and its linkage with sustainability is essential. Therefore, the economic value of WEPs must be highlighted and studied, in which edible flowers can play a key role; they can be prepared for sale in different formats, such as fresh, dried, or even candied. This variability together with the color of these flowers makes them highly attractive and a good business proposition within the flowers market. Globalization and online marketing have created an emerging market for this type of product worldwide. The sale of these flowers is usually in punnets of between 6 and 15 flowers, depending on the type of flower and the season. The price of these punnets also depends on the type of flower, but is usually between 8 and 17 euros, some of them reaching approximately 40 euros [85,86,87,88]. In recent years, haute cuisine has valued this type of flowers, either as a decoration on dishes, or in search of new flavors, aromas or appearance. One of the best restaurants in the world is “Mugaritz” with two Michelin stars and directed by Andoni Luis Aduriz; this restaurant has been innovative and pioneering when it comes to basing its dishes on wild plants, also using edible flowers.

Apart from this direct way of selling edible flowers, there are also lollipops with edible flowers inside, or crystallized. The price range is between 21 and 60 euros [85].

Other ways in which these plants contribute their economic value is through disclosure through books. In 2004, the book “Clorofilia” by Andoni Luis Aduriz was released, where information was collected on 50 wild plants, their flowering calendar, taxonomic information and recipes [89]. Another informative book on wild plants and herbs was published by the Basque Culinary Center, which contains detailed information on 180 varieties of wild plants/herbs from a botanical and culinary point of view [90].

So far, we have studied the entire market for WEPs, either directly or through other sources; however, for many families these plants are their livelihood. In the study by Mokria et al. [91], the need to promote strategic plans at the national level in Ethiopia for the sustainable use and domestication of these plants is highlighted. This would aim at socio-economic improvement and to help in achieving one of the SDG targets (2. End hunger and malnutrition). In northeast India, WEPs are critical to the survival of ethnic communities. A survey was conducted among 30 local vendors and 550 households. The results were overwhelming, registering, in consumption or sale, five wild edible mushrooms and 158 wild plants (78.8% of them being edible). In most households, wild plants influenced family income, accounting for between 5 and 75% of family income. All these results clearly demonstrated the importance of these wild plants for the subsistence and survival of many rural communities [92].

Not only are these plants important in ethnic communities, but the study carried out by Matsuura [93] showed their importance for the Japanese population. This study indicated that, in rural areas near Fukushima, it is not possible to harvest WEPs or edible mushrooms, which precludes a complete diet full of all the essential traditional products. The results concluded that the collection rate should remain very low for a few years within a radius of between 12 and 30 km from Fukushima (Kawauchi village, where the study was carried out) due to safety reasons. These data show the importance in these communities for their livelihood.

With all these data, the economic importance of WEPs is evident, being essential for many families around the world to survive.

## 10. Conclusions

The topic of this review is a step into the future, because developing countries are facing a serious problem, the fast growth of their population and the consequent increase food needs/availability. Furthermore, in many rural communities it is a necessity, because WEPs are not only a food but a way of earning a living. WEPs are a natural source of minerals, vitamins, fiber, and antioxidants, and at the same time they are inexpensive, as they are not cultivated. Therefore, they are a real alternative in trying to reduce this gap between food production and demand, especially to produce natural and sustainable food additives, such as flavorings and aromas. In addition, the food industry could take advantage of the properties of WEPs to develop nutritional, organic, and sustainable foods. Nevertheless, more research is needed to go beyond just their composition information (which is the most commonly found in the scientific databases for this type of plant); information on their technological and functional properties is needed to include them in industrial processes. Toxicological studies are also needed to determine their activities (e.g., anti,-mutagenic, etc.). Thus, a deep investigation into WEPs is needed to start developing commercial products based on these inexpensive and sustainable plants/ingredients. Sustainability, nutrition, and the agri-food industry converge around these plants.

## Figures and Tables

**Table 2:** Sugars reported in wild edible plants, WEPs.

Plant Species	Part of Plant	Unit	Sugars			Reference
			Fructose	Glucose	Sucrose	
*Asparagus acutifolius* L.	Stems	g/100 g dw	2.49 ± 0.13	1.98 ± 0.04	4.27 ± 0.12	[32]
*Borago officinalis* L.	Leaves	g/100 g dw	0.14 ± 0.03	0.58 ± 0.06	1.52 ± 0.13	[30]
*Bryonia dioica* Jacq.	Stems	g/100 g dw	3.45 ± 0.08	2.97 ± 0.09	0.572 ± 0.014	[32]
*Chenopodium ambrosioides* L.	Leaves	g/100 g dw	0.24 ± 0.01	0.46 ± 0.01	1.43 ± 0.12	[54]
*Foeniculum vulgare* Mill.	Leaves	g/100 g fw	0.49 ± 0.05	0.76 ± 0.12	0.04 ± 0.00	[55]
*Glechoma hederacea* L.	Leaves	g/100 g fw	0.15 ± 0.01	0.08 ± 0.02	0.40 ± 0.06	[27]
*Helichrysum stoechas* (L.) Moench	Stems	g/100 g dw	1.02 ± 0.04	0.59 ± 0.02	1.84 ± 0.09	[56]
*Malva sylvestris* L.	Flowers	g/100 g dw	8.72 ± 0.14	7.36 ± 0.13	2.74 ± 0.05	[36]
*Mentha pulegium* L.	Inflorescences	g/100 g dw	2.39 ± 0.11	3.37 ± 0.22	4.62 ± 0.28	[37]
*Montia fontana* subsp. *amporitana* Sennen	Leaves	g/100 g dw	0.76 ± 0.17	1.00 ± 0.02	0.44 ± 0.05	[30]
*Nasturtium officinale* R. Br.	Leaves	mg/kg	1104 ± 31	696 ± 20	233 ± 51	[39]
*Origanum vulgare* L.	Leaves	g/100 g fw	0.19 ± 0.01	0.58 ± 0.01	0.30 ± 0.00	[27]
*Portulaca oleracea* L.	Leaves	mg/100 g fw	290 (202–352) ^‡^	81.25 (59–118)	161.5 (75–271)	[57]
*Pterospartum tridentatum* (L.) Willk.	Flowers	g/100 g dw	3.49 ± 0.11	1.19 ± 0.05	0.58 ± 0.03	[41]
*Rubus ulmifolius* Schott	Flowers	g/100 g dw	1.66 ± 0.21	2.23 ± 0.19	1.34 ± 0.15	[56]
*Rumex acetosella* L.	Leaves	g/100 g dw	0.60 ± 0.00	0.73 ± 0.01	0.21 ± 0.07	[30]
*Rumex induratus* Boiss. & Reut	Leaves	g/100 g dw	1.71 ± 0.09	1.26 ± 0.20	1.25 ± 0.31	[30]
*Raphanus raphanistrum* L.	Leaves	g/100 g fw	0.153 ± 0.004	0.348 ± 0.003	-	[42]
*Tamus communis* L.	Leaves	g/100 g dw	3.83 ± 0.13	1.80 ± 0.14	0.695 ± 0.05	[32]
*Thymus mastichina* L.	Leaves	g/100 g fw	0.45 ± 0.01	0.97 ± 0.11	0.02 ± 0.00	[27]
*Thymus pulegioides* L.	Inflorescences	g/100 g dw	0.22 ± 0.00	0.33 ± 0.03	1.06 ± 0.02	[37]
*Umbilicus rupestris* (Salisb.) Dandy	Leaves	g/100 g fw	-	-	0.082 ± 0.002	[46]

^‡^ Mean value (minimum-maximum).

**Table 3:** Fatty acids reported in wild edible plants, WEPs.

Plant Species	Part of Plant	Unit	C16:0 (Palmitic Acid)	C18:1 (Oleic Acid)	C18:2 (Linoleic Acid)	C18:3 (Linolenic Acid)	Reference
*Allium ampeloprasum* L.	Bulbs	%	26.42 ± 0.30	7.39 ± 0.42	53.45 ± 0.27	nd	[67]
*Anchusa azurea* Mill.	Leaves	%	10.45 ± 0.62	2.20 ± 0.00	12.16 ± 0.11	64.74 ± 0.23	[69]
*Apium nodiflorum* (L.) Lag	Stems	%	16.29 ± 0.96	3.33 ± 0.02	24.60 ± 0.77	43.46 ± 0.08	[70]
*Asparagus acutifolius* L.	Stems	%	17.5 ± 0.2	4.94 ± 0.35	44.5 ± 1.3	23.7 ± 0.9	[32]
*Bellis perennis* L.	Aerial parts	%	1.5	-	5.1	13.6	[71]
*Beta maritima* L.	Leaves	%	11.03 ± 0.15	3.51 ± 0.01	21.28 ± 0.04	57.80 ± 0.03	[70]
*Borago officinalis* L.	Leaves	%	12.03 ± 0.70	2.08 ± 0.20	9.50 ± 1.25	12.26 ± 1.90	[30]
*Bryonia dioica* Jacq.	Stems	%	13.5 ± 0.3	1.52 ± 0.09	6.39 ± 0.16	70.3 ± 0.1	[32]
*Cichorium intybus* L.	Leaves	%	10.64 ± 0.63	1.61 ± 0.04	21.14 ± 0.06	60.45 ± 0.41	[70]
*Chenopodium ambrosioides* L.	Leaves	%	14.16 ± 0.03	6.90 ± 0.12	19.23 ± 0.12	48.54 ± 0.13	[54]
*Chondrilla juncea* L.	Leaves	%	12.96 ± 0.47	1.91 ± 0.01	19.92 ± 0.17	56.27 ± 0.13	[70]
*Daucus carota* L.	Roots	%	tr	-	1.0	-	[71]
*Diplotaxis erucoides* (L.) DC	Aerial parts	%	18.23	3.42	39.31 ^‡^	-	[11]
*Diplotaxis virgata* (Cav.) DC	Flowers	%	14.35	-	36.01 ^‡^	-	[11]
*Foeniculum vulgare* Mill.	Leaves	%	20.15 ± 0.09	4.35 ± 0.37	23.25 ± 0.07	43.55 ± 0.40	[55]
*Glechoma hederacea* L.	Leaves	%	12.23 ± 0.23	35.12 ± 0.27	8.15 ± 0.08	27.87 ± 0.20	[27]
*Helichrysum stoechas* (L.) Moench	Stems	%	13.24 ± 0.16	6.15 ± 0.79	25.67 ± 0.08	22.79 ± 1.86	[56]
*Humulus lupulus* L.	Leaves	%	19.52 ± 0.61	1.88 ± 0.10	29.72 ± 0.85	38.16 ± 0.02	[70]
*Malva sylvestris* L.	Flowers	%	9.79 ± 1.07	3.31 ± 0.42	11.96 ± 0.42	67.79 ± 0.96	[36]
*Mentha pulegium* L.	Inflorescences	%	14.82 ± 0.09	5.77 ± 0.20	16.27 ± 0.33	37.00 ± 0.35	[37]
*Montia fontana* subsp. *amporitana* Sennen	Leaves	%	17.22 ± 1.06	2.37 ± 0.38	18.71 ± 0.45	55.57 ± 0.80	[30]
*Origanum vulgare* L.	Leaves	%	4.95 ± 0.10	5.08 ± 0.01	23.22 ± 0.14	62.34 ± 0.04	[27]
*Papaver rhoeas* L.	Leaves	%	9.66 ± 0.39	1.36 ± 0.00	16.53 ± 0.01	64.98 ± 0.07	[70]
*Portulaca oleracea* L.	Leaves	%	24.7 (23.4–26.9) ^¥^	12.4 (9.7–15.1)	28.8 (25.1–32.9)	23.6 (17.9–28.4)	[57]
*Pterospartum tridentatum* (L.) Willk.	Flowers	%	14.84 ± 0.83	9.22 ± 1.09	19.59 ± 0.67	29.50 ± 1.98	[41]
*Rumex acetosella* L.	Leaves	%	11.23 ± 0.73	3.43 ± 0.32	20.18 ± 0.48	51.34 ± 1.41	[30]
*Rumex induratus* Boiss. & Reut	Leaves	%	9.36 ± 0.71	2.20 ± 0.05	13.76 ± 0.01	58.84 ± 1.03	[30]
*Rumex papillaris* Boiss. and Reut.	Leaves	%	11.20 ± 0.32	5.80 ± 0.14	22.79 ± 0.19	51.77 ± 0.14	[70]
*Rumex pulcher* L.	Leaves	%	9.30 ± 0.11	4.22 ± 0.01	17.03 ± 0.16	62.97 ± 0.03	[70]
*Raphanus raphanistrum* L.	Leaves	mg/100 g fw ^¶^	25.2 ± 2.3	2.4 ± 0.3	24 ± 2	171 ± 16	[42]
*Sambucus nigra* L.	Aerial parts	%	tr	-	16.1	2.6	[71]
*Scolymus hispanicus* L.	Leaves	%	20.65 ± 0.85	6.41 ± 0.07	26.44 ± 0.26	30.55 ± 0.23	[70]
*Silene vulgaris* (Moench) Garcke	Leaves	%	13.5 (13.1–15.1)	2.4 (2.1–2.7)	22.4 (18.9–24.4)	54.5 (51.2–56.9)	[43]
*Silybum marianum* (L.) Gaertn.	Leaves	%	28.69 ± 1.60	3.86 ± 0.10	31.01 ± 0.63	21.60 ± 0.25	[70]
*Sonchus oleraceus* L.	Leaves	%	10.43 ± 0.70	0.92 ± 0.10	13.78 ± 0.61	nd	[70]
*Tamus communis* L.	Leaves	%	17.0 ± 0.7	7.51 ± 0.18	42.0 ± 0.3	27.5 ± 0.4	[32]
*Taraxacum obovatum* (Willd.) DC.	Leaves	%	11.83 ± 0.09	3.24 ± 0.01	17.64 ± 0.08	58.53 ± 0.23	[70]
*Thymus mastichina* L.	Leaves	%	10.22 ± 0.20	9.82 ± 0.18	11.83 ± 0.06	45.65 ± 0.55	[27]
*Thymus pulegioides* L.	Inflorescences	%	16.70 ± 0.22	11.40 ± 0.10	12.98 ± 0.52	36.69 ± 0.25	[37]
*V. x Wittrockiana*	Flowers	g/100 g dw ^£^	36.41	8.27	32.30	nd	[4]
*Umbilicus rupestris* (Salisb.) Dandy	Leaves	%	10.6 ± 0.8	0.641 ± 0.002	18.3 ± 0.6	62 ±2	[46]

^‡^ Linoleic acid C18: 2 y Linolelaidic acid C18:2; nd = not detected; tr = trace; ^¥^ Mean value (minimum-maximum); ^£^ dw = dry weight; ^¶^ fw = fresh weight.

**Table 4:** Total phenolic content (TPC) and total flavonoids content (TFC) in wild edible plants.

Plant Species	Part of Plants	Unit TPC	TPC	Unit TFC	TFC	Reference
*Asystasia gangetica* (L.) T.Anderson	-	mg GAE/g de ^‡^	91.797 ± 0.295	mg RE/g de ^§^	20.132 ± 0.093	[31]
*Achyranthes aspera* L.	-	mg GAE/g de	74.831 ± 0.243	mg RE/g de	20.793 ± 0.122	[31]
*Ageratum conyzoides* (L.) L.	Flowers	mg GAE/g	4.63 ± 0.52	-	-	[81]
*Allamanda cathartica* L.	Flowers	mg GAE/g	4.16 ± 0.11	-	-	[81]
*Allium ampeloprasum* L.	Bulbs	mg GAE/g	5.70 ± 0.62	mg CE/g ^ø^	0.86 ± 0.5	[67]
*Amaranthus viridis* L.	-	mg GAE/g de	50.700 ± 0.079	mg RE/g de	19.970 ± 0.252	[31]
*Anagallis arvensis* (L.)	Aerial parts	mg GAE/g	27.54 ± 0.92	mg QE/g ^£^	26.15 ± 0.85	[84]
*Anchusa azurea* Mill.	Leaves	mg GAE/g extract.	148.62 (146.62–150.62) ^¥^	mg CE/g extract.	84.81 (80.78–88.84)	[72]
*Apium nodiflorum* (L.) Lag.	Leaves	mg GAE/g extract.	80.47 ± 4.41	mg CE/g extract.	45.48 ± 1.61	[69]
*Asparagus acutifolius* L.	Stems	mg GAE/g extract.	624 ± 28	mg CE/g extract.	57.8 ± 2.4	[32]
*Asparagus acutifolius* L.	Leaves	mg CAE/kg ww ^¶^	43.1	mg QE/kg ww	2262.9	[83]
*Bahuinia purpurea L.*	Flowers	mg GAE/g	6.14 ± 0.30	-	-	[81]
*Beta marítima* L.	Leaves	mg GAE/g extract.	61.91 ± 7.51	mg CE/g extract.	21.55 ± 0.87	[72]
*Berberis aristata* DC.	Leaves	mg GAE/g dw	135.56 ± 3.26	mg QE/g dw	82.05 ± 0.78	[28]
*Bidens pilosa L.*	Flowers	mg GAE/g	8.12 ± 0.41		-	[81]
*Blumea lacera* (Burm. f.) DC.	Leaves	mg GAE/g dw	95.23 ± 1.35	mg QE/g dw	59.87 ± 0.93	[28]
*Bombax malabaricum* DC.	Flowers	mg GAE/g	3.88 ± 0.17	-	-	[81]
*Borago officinalis* L.	Leaves	mg CAE/kg ww	64.0	mg QE/kg ww	189.4	[83]
*Bougainvillea spectabilis* Willd.	Flowers	mg GAE/g	6.87 ± 0.23	-	-	[81]
*Brassica campestris* L.	Flowers	mg GAE/g	3.32 ± 0.09	-	-	[81]
*Brunfelsia acuminata* (Pohl) Benth.	Flowers	mg GAE/g	4.08 ± 0.25	-	-	[81]
*Bryonia dioica* Jacq.	Stems	mg GAE/g extract.	258 ± 22	mg CE/g extract.	18.1 ± 1.2	[32]
*Calliandra haematocephala* Hassk.	Flowers	mg GAE/g	14.43 ± 0.71	-	-	[81]
*Camellia japonica* L.	Flowers	mg GAE/g	5.14 ± 0.29	-	-	[81]
*Chaenomeles sinensis* (Thouin) Koehne	Flowers	mg GAE/g	13.93 ± 0.34	-	-	[81]
*Chrysanthemum coronarium* L.	Flowers	mg GAE/g	3.76 ± 0.29	-	-	[81]
*Chrysanthemum morifolium* Ramat	Flowers	mg GAE/g	3.75 ± 0.11	-	-	[81]
*Cichorium intybus* L.	Leaves	mg CAE/kg ww	158.6	mg QE/kg ww	1066.0	[83]
*Chenopodium ambrosioides* L.	Leaves	mg GAE/100 g dw	822.33 ± 12.25	mg CE/g extract.	768.27 ± 10.70	[54]
*Chondrilla juncea* L.	Leaves	mg GAE/g extract.	37.66 ± 2.44	mg CE/g extract.	7.43 ± 0.28	[72]
*Dianthus caryophyllus* L.	Flowers	mg GAE/g	5.50 ± 0.28	-	-	[81]
*Dianthus chinensis* L.	Flowers	mg GAE/g	5.27 ± 0.25	-	-	[81]
*Diplotaxis erucoides* DC.	Leaves	mg CAE/kg ww	48.7	mg QE/kg ww	2876.7	[83]
*Enhydra fluctuans* Lour.	-	mg GAE/g de	70.338 ± 0.103	mg RE/g de	21.759 ± 0.039	[31]
*Erythrina variegata* L.	Leaves	mg GAE/g dw	170.33 ± 2.18	mg QE/g dw	53.42 ± 1.23	[28]
*Erythrina variegata* L.	Flowers	mg GAE/g	3.90 ± 0.29	-	-	[81]
*Foeniculum vulgare* Mill.	Leaves	mg GAE/g meth. extract.	42.16 ± 0.98	mg CE/g extract.	9.72 ± 0.70	[69]
*Gerbera jamosenii* Bolus ex Hook.f.	Flowers	mg GAE/g	4.89 ± 0.15	-	-	[81]
*Gladiolus x hybridus* C.Morren	Flowers	mg GAE/g	2.30 ± 0.23	-	-	[81]
*Glechoma hederacea* L.	Leaves	mg GAE/g	196.61 ± 6.09	mg CE/g	95.02 ± 2.73	[80]
*Helianthus annuus* L.	Flowers	mg GAE/g	1.86 ± 0.22	-	-	[81]
*Helichrysum stoechas* (L.) Moench	Inflorescences	mg GAE/g	184.42 ± 0.35	mg CE/g	34.75 ± 0.83	[56]
*Hibiscus rosa-sinensis* L.	Flowers	mg GAE/g	6.80 ± 0.63	-	-	[81]
*Humulus lupulus* L.	Leaves	mg GAE/g	55.83 ± 1.34	mg CE/g	9.56 ± 0.65	[69]
*Hygrophilla schulli (Hamilt.)* M.R.Almeida & S.M.Almeida	Leaves	mg GAE/g dw	148.05 ± 3.21	mg QE/g dw	87.12 ± 0.86	[28]
*Impatiens walleriana* Hook.f.	Flowers	mg GAE/g	7.62 ± 0.16	-	-	[81]
*Ipomoea aquatica* Forssk.	-	mg GAE/g de	45.449 ± 0.130	mg RE/g de	13.941 ± 0.040	[31]
*Ipomoea cairica* (L.) Sweet	Flowers	mg GAE/g	1.77 ± 0.13	-	-	[81]
*Iris japonica* Thunb.	Flowers	mg GAE/g	0.63 ± 0.03	-	-	[81]
*Jasminum nudiflorum* Lindl.	Flowers	mg GAE/g	3.08 ± 0.09	-	-	[81]
*Jatropha integerrima* Jacq.	Flowers	mg GAE/g	17.22 ± 0.77	-	-	[81]
*Lantana camara* L.	Flowers	mg GAE/g	3.50 ± 0.08	-	-	[81]
*Ligustrum sinense* Lour.	Flowers	mg GAE/g	6.22 ± 0.11	-	-	[81]
*Lilium brownii* F.E.Br. ex Miellez	Flowers	mg GAE/g	1.27 ± 0.13	-	-	[81]
*Limonium sinuatum* (L.) Mill.	Flowers	mg GAE/g	34.17 ± 1.17	-	-	[81]
*Loropetalum chinense* var. *rubrum* Yieh	Flowers	mg GAE/g	11.46 ± 0.26	-	-	[81]
*Magnolia soulangeana* Soul.-Bod.	Flowers	mg GAE/g	5.30 ± 0.22	-	-	[81]
*Malva sylvestris* L.	Flowers	mg GAE/g extract.	386.45 ± 8.54	mg CE/g extract.	210.81 ± 7.99	[36]
*Malvaviscus arboreus* Cav.	Flowers	mg GAE/g	3.12 ± 0.41	-	-	[81]
*Matthiola incana* (L.) R.Br.	Flowers	mg GAE/g	1.70 ± 0.08	-	-	[81]
*Mentha pulegium* L.	Inflorescences	mg GAE/g extract.	331.69 ± 19.63	mg CE/g extract.	139.85 ± 1.27	[37]
*Montia fontana* L.	Leaves	mg GAE/g extract.	75.53 ± 7.05	mg CE/g extract.	16.67 ± 0.62	[69]
*Nasturtium officinale* R. Br.	Aerial parts	g GAE/kg extract	87 ± 2	g CE/kg extract	36 ± 1	[39]
*Oldenlandia corymbosa* Aiton	-	mg GAE/g de	47.184 ± 0.060	mg RE/g de	15.848 ± 0.125	[31]
*Oncidium varicosum* Lindl.	Flowers	mg GAE/g	4.46 ± 0.40	-	-	[81]
*Origanum vulgare* subsp. *virens*	Inflorencences	mg GAE/g	368.58 ± 18.18	mg CE/g	224.15 ± 0.96	[80]
*Orostachys fimbriata* (Turcz.) A. Berger	Flowers	mg GAE/g	12.36 ± 0.43	-	-	[81]
*Osmanthus fragrans* Lour.	Flowers	mg GAE/g	16.00 ± 0.57	-	-	[81]
*papver corymbosa* DC.	Flowers	mg GAE/g	2.20 ± 0.07	-	-	[81]
*Papaver rhoeas* L.	Leaves	mg GAE/g extract.	25.86 ± 3.52	mg CE/g extract.	12.00 ± 0.46	[72]
*Pelargonium hortorum* L.H. Bailey	Flowers	mg GAE/g	25.68 ± 1.02	-	-	[81]
*Phaseolus vulgaris* L.	Flowers	mg GAE/g	1.86 ± 0.10	-	-	[81]
*Platydocon grandiflorus* (Jacq.) A.DC.	Flowers	mg GAE/g	4.57 ± 0.28	-	-	[81]
*Portulaca oleracea* L.	Leaves	mg GAE/g extract.	12.89 (7.65–20.1)	mg CE/g extract.	1.76 (0.12–5.30)	[57]
*Pterospartum tridentatum* (L.) Willk.	Flowers	mg ClAE/g extract.	523.42 ± 36.09	mg QE/g extract.	58.12 ± 5.78	[41]
*Rhapniolepis indica* (L.) Lindl.	Flowers	mg GAE/g	7.97 ± 0.29	-	-	[81]
*Rhododendron simsii* Planch	Flowers	mg GAE/g	6.75 ± 0.22	-	-	[81]
*Rhoeo discolor* (L’Hér.) Hance	Flowers	mg GAE/g	2.56 ± 0.05	-	-	[81]
*Rubus ulmifolius* Schott	Flowers	mg GAE/g extract.	257.89 ± 3.28	mg CE/g extract.	172.45 ± 3.42	[56]
*Rumex acetosella* L.	Leaves	mg GAE/g extract.	141.58 ± 3.67	mg CE/g extract.	67.91 ± 3.02	[30]
*Rumex induratus* Boiss. & Reut.	Leaves	mg GAE/g extract.	117.08 ± 2.54	mg CE/g extract.	89.78 ± 2.81	[30]
*Rumex papillaris* Boiss. & Reut.	Leaves	mg GAE/g extract.	104.18 ± 4.17	mg CE/g extract.	39.49 ± 3.26	[72]
*Rumex pulcher* L.	Leaves	mg GAE/g extract.	73.44 ± 5.32	mg CE/g extract.	26.14 ± 0.87	[72]
*Salvia splendens* Sellow ex Roem. & Schult.	Flowers	mg GAE/g	2.57 ± 0.07	-	-	[81]
*Sesbania sesban* (L.) Merr.	Leaves	mg GAE/g dw	167.66 ± 2.37	mg QE/g dw	97.16 ± 1.38	[28]
*Silene vulgaris* (Moench) Garcke	Leaves	mg GAE/g extract.	26.72 ± 1.63	mg CE/g extract.	21.65 ± 5.53	[69]
*Silybum marianum* (L.) Gaertn.	Leaves	mg GAE/g extract.	3.72 ± 0.36	mg CE/g extract.	1.13 ± 0.27	[72]
*Sinapis incana* (L.) Maly	Leaves	mg CAE/kg ww	92.2	mg QE/kg ww	1364.7	[83]
*Sinapis nigra* (L.) W.D.J.Koch	Leaves	mg CAE/kg ww	44.3	mg QE/kg ww	1545.6	[83]
*Sonchus oleraceus* L.	Leaves	mg GAE/g extract.	51.33 ± 1.75	mg CE/g extract.	14.83 ± 0.98	[72]
*Sophora viciifolia* Hance	Flowers	mg GAE/g dry extract	143.8 ± 8.7	mg RE/g dry extract	237.2 ± 10.3	[5]
*Strelitzia reginae* Banks ex Aiton	Flowers	mg GAE/g	9.40 ± 0.58	-	-	[81]
*Tamus communis* L.	Leaves	mg GAE/g extract.	49.51 ± 4.07	mg CE/g extract.	9.33 ± 1.44	[69]
*Taraxacum obovatum* (Willd.) DC.	Leaves	mg GAE/g extract.	58.26 ± 0.90	mg CE/g extract.	30.03 ± 0.66	[72]
*Thymus mastichina* L.	Inflorescences	mg GAE/g	165.29 ± 1.11	mg CE/g	83.85 ± 1.42	[80]
*Thymus pulegioides* L.	Inflorescences	mg GAE/g extract.	210.49 ± 21.16	mg CE/g extract.	128.24 ± 6.00	[37]
*Viola x Wittrockiana*	Flowers	mg GAE/g	6.08	-	-	[4]
*Wedelia trilobata* (L.) Hitchc.	Flowers	mg GAE/g	3.85 ± 0.03	-	-	[81]
*Youngia japonica* (L.) DC.	Flowers	mg GAE/g	1.11 ± 0.03	-	-	[81]
*Zantedeschia aethiopica* (L.) Spreng	Flowers	mg GAE/g	3.07 ± 0.07	-	-	[81]

^¥^ Mean value (minimum-maximum); ^‡^ GAE = gallic acid equivalents; ^§^ RE = rutin equivalents; ^ø^ CE = catequin equivalents; ^£^ QE = quercetin equivalent; ^¶^ CAE = caffeic acid equivalent; ClAE = chlorogenic acid equivalents.

## Data Availability

Not applicable.

## Supplementary Materials

The following supporting information can be downloaded at: https://www.mdpi.com/article/10.3390/foods12051012/s1, Table S1: List of Wild Edible Plants, WEPs; Table S2: Organic acids content in Wild Edible Plants, WEPs; Table S3: Daily value of minerals recommended by Food & Drug Administration (FDA); Table S4: Mineral elements in Wild Edible Plants, WEP’s. [25,34,35,38,42,46,57,61,66,67,68]
