# Supplementary material for: Valorization of Wild Edible Plants as Food Ingredients and Their Economic Value
Source: Foods. 2023 Feb 27;12(5):1012. doi: 10.3390/foods12051012 (PMC10001278; doi:10.3390/foods12051012)
Supplement: Supplementary file 1 [file foods-12-01012-s001.zip › foods-2237588-supplementary.pdf]

Table S1. List of Wild Edible Plants, WEPs.

Wild Edible Plant	Family	Wild Edible Plant	Family
Asystasia ganjetica (L.) T.Anderson	Acanthaceae	*Orostachys fimbriata* (Turcz.) A. Berger	Crassulaceae
Hygrophila schulli (Hamilt.) M.R. Almeida & S.M. Almeida	Acanthaceae	*Umbilicus rupestris* (Salisb.) Dandy	Crassulaceae
Sambucus nigra L.	Adoxaceae	*Bryonia dioica* Jacq.	Cucurbitaceae
Achyranthes aspera L.	Amaranthaceae	*Tamus communis* L.	Dioscoreaceae
Amaranthus viridis L.	Amaranthaceae	*Rhododendron simsii* Planch	Ericaceae
Beta maritima L.	Amaranthaceae	*Jatropha integerrima* Jacq.	Euphorbiaceae
Chenopodium ambrosioides L.	Amaranthaceae	*Bauhinia purpurea* L.	Fabaceae
Allium ampeloprasum L.	Amaryllidaceae	*Calliandra haematocephala* Hassk.	Fabaceae
Apium nodiflorum (L.) Lag.	Apiaceae	*Erythrina variegata* L.	Fabaceae
Daucus carota L.	Apiaceae	*Phaseolus vulgaris* L.	Fabaceae
Foeniculum vulgare Mill.	Apiaceae	*Pterospartum tridentatum* (L.) Willk.	Fabaceae
Allamanda cathartica L.	Apocynaceae	*Sesbania sesban* (L.) Merr	Fabaceae
Zantedeschia aethiopica (L.) Spreng	Araceae	*Sophora viciifolia* Hance	Fabaceae
Asparagus acutifolius	Asparagaceae	*Pelargonium hortorum* L.H. Bailey	Geraniaceae
Ageratum conyzoides (L.) L.	Asteraceae	*Loropetalum chinnense* var. *Rubrum* Yieh	Hamamelidaceae
Bellis perennis L.	Asteraceae	*Gladiolus x hybridus* C.Morren	Iridaceae
Bidens pilosa L.	Asteraceae	*Iris japonica* Thunb.	Iridaceae
Blumea lacera (Burm. f.) DC.	Asteraceae	*Glechoma hederacea* L.	Lamiaceae
Cynara cardunculus L.	Asteraceae	*Mentha pulegium* L.	Lamiaceae
Chondrilla juncea L.	Asteraceae	*Origanum vulgare* L.	Lamiaceae
Chrysanthemum coronarium L.	Asteraceae	*Origanum vulgare* subsp. *virens*	Lamiaceae
Cichorium intybus L.	Asteraceae	*Salvia splendens* Sellow ex Roem. & Schult.	Lamiaceae
Enhydra fluctuans Lour.	Asteraceae	*Thymus mastichina* L.	Lamiaceae
Gerbera jamesonii Bolus ex Hook.f.	Asteraceae	*Thymus pulegioides* L.	Lamiaceae
Helianthus annuus L.	Asteraceae	*Lilium brownii* F.E.Br. ex Miellez	Liliaceae
Helichrysum stoechas (L.) Moench.	Asteraceae	*Magnolia soulangeana* Soul.-Bod.	Magnoliaceae
Hymenonema graecum (L.) DC	Asteraceae	*Bombax malabaricum* DC.	Malvaceae
Picris echioides L.	Asteraceae	*Hibiscus rosa-sinensis* L.	Malvaceae
Reichardia picroides (L.) Roth	Asteraceae	*Malva sylvestris* L.	Malvaceae
Scolymus hispanicus L.	Asteraceae	*Malvaviscus arboreus* Cav.	Malvaceae
Silybum marianum (L.) Gaertn.	Asteraceae	*Montia fontana* L.	Montiaceae

Sonchus asper L.	Asteraceae
Sonchus oleraceus L.	Asteraceae
Sonchus tenerrimus L.	Asteraceae
Taraxacum obovatum (Willd.) DC	Asteraceae
Taraxacum officinale Webb	Asteraceae
Urospermum picroides (L.) Scop. ex F.W.Schmidt	Asteraceae
Wedelia trilobata (L.) Hitchc.	Asteraceae
Youngia japónica (L.) DC.	Asteraceae
Impatiens walleriana Hook.f.	Balsaminaceae
Berberis aristata DC.	Berberidaceae
Anchusa azurea Mill.	Boraginaceae
Borago officinalis L.	Boraginaceae
Brassica campestris L.	Brassicaceae
Diplotaxis erucoides (L.) DC.	Brassicaceae
Diplotaxis tenuifolia (L.) DC.	Brassicaceae
Diplotaxis virgata (Cav.) DC	Brassicaceae
Matthiola incana (L.) R.Br.	Brassicaceae
Nasturtium officinale R. Br.	Brassicaceae
Raphanus raphanistrum L.	Brassicaceae
Sinapis incana (L.) Maly	Brassicaceae
Sinapis nigra (L.) W.D.J.Koch	Brassicaceae
Platycodon grandiflorus (Jacq.) A.DC.	Campanulaceae
Humulus lupulus L.	Cannabaceae
Dianthus caryophyllus L.	Caryophyllaceae
Dianthus chinensis L.	Caryophyllaceae
Silene vulgaris (Moench) Garcke	Caryophyllaceae
Rhoeo discolor (L'Hér.) Hance	Commelinaceae
Ipomoea aquatica Forssk.	Convolvulaceae
Ipomoea cairica (L.) Sweet	Convolvulaceae
Montia fontana subsp. *amporitana* Sennen	Montiaceae
Bougainvillea spectabilis Willd.	Nyctaginaceae
Jasminum nudiflorum Lindl.	Oleaceae
Ligustrum sinense Lour.	Oleaceae
Osmanthus fragrans Lour.	Oleaceae
Oncidium varicosum Lindl.	Orchidaceae
Oxalis corymbosa DC.	Oxalidaceae
Papaver rhoeas L.	Papaveraceae
Limonium sinuatum (L.) Mill.	Plumbaginaceae
Rumex acetosella L.	Polygonaceae
Rumex induratus Boiss. & Reut.	Polygonaceae
Rumex papillaris Boiss. & Reut.	Polygonaceae
Rumex pulcher L.	Polygonaceae
Portulaca oleracea L.	Portulacaceae
Anagallis arvensis (L.)	Primulaceae
Chaenomeles sinensis (Thouin) Koehne	Rosaceae
Rhapniolepis indica (L.) Lindl.	Rosaceae
Rubus ulmifolius Schott	Rosaceae
Oldenlandia corymbosa Aiton	Rubiaceae
Brunfelsia acuminata (Pohl) Benth.	Solanaceae
Strelitzia reginae Banks ex Aiton	Strelitziaceae
Camellia japónica L.	Theaceae
Lantana cámara L.	Verbenaceae
Viola x wittrockiana	Violaceae

Table S2. Organic acids content in Wild Edible Plants, WEPs.

Plant species	Part of plants	Oxalic (mg/ 100 g)	Malic (mg/ 100 g)	Shikimic (mg/ 100 g)	References
Hymenonema graecum (L.) DC	Leaves	972	110	244	[61]
Picris echioides L.	Leaves	562	105	87	[61]
Portulaca oleracea L.	Aerial parts	554	60,25	0	[57]
Reichardia picroides (L.) Roth	Leaves	625	91	2,22	[61]
Raphanus raphanistrum L.	Leaves	706	580	0	[42]
Sonchus oleraceus L.	Leaves	777	415	167	[61]
Taraxacum officinale Webb	Leaves	386	220	53	[61]
Umbilicus rupestris (Salisb.) Dandy	Leaves	480	210	0	[46]
Urospermum picroides (L.) Scop. ex F.W.Schmidt	Leaves	574	248	75	[61]

Table S3. Daily value of minerals recommended by Food & Drug Administration (FDA).

Mineral	Daily Value†
Calcium	1300 mg
Chloride	2300 mg
Chromium	35 µg
Copper	0.9 mg
Iodine	150 µg
Iron	18 mg
Magnesium	420 mg
Manganese	2.3 mg
Molybdenum	45 µg
Phosphorus	1250 mg
Potassium	4700 mg
Selenium	55 µg
Sodium	2300 mg
Zinc	11 mg

† The Daily Values are reference amounts of nutrients to consume or not to exceed each day; the table is based on data from the Food & Drug Administration (FDA) https://www.accessdata.fda.gov/scripts/InteractiveNutritionFactsLabel/assets/InteractiveNFL_Vitamins&MineralsChart_October2021.pdf (accessed on 12 September 2022)

Table S4. Mineral elements in Wild Edible Plants, WEP's.

Plant species	Part of plants	Unit	Na	K	Ca	Mg	Cu	Fe	Mn	Zn	Reference
Allium ampeloprasum L.	Bulbs	mg/100 g	55 (44-67)[¥]	439 (147-533)	70 (30-82)	14 (9-16)	0.11 (0.05-0.22)	0.60 (0.20-0.92)	0.11 (0.06-0.15)	0.75 (0.03-1.67)	[67]
Beta maritima L.	Leaves	mg/100 g	207 (45-288)	1223 (597-2356)	96 (19-250)	73 (13-136)	0.21 (0.09-0.35)	2.24 (1.42-3.97)	0.82 (0.57-1.23)	0.88 (0.64-1.26)	[66]
Borago officinalis L.	Leaves	mg/ kg fw	637.1±45.8	5219.4±489.7	1686.4±76.3	385.5±20.4	-	-	-	-	[68]
Cichorium intybus L.	Leaves	mg/100 g	75.9 (37.2–170)	699 (535–1085)	100 (45–136)	25.5 (9.8–33.9)	0.11 (0.06-0.21)	1.17 (0.41–2.00)	0.26 0.17–0.47	0.37 (0.08–0.51)	[25]
Cichorium intybus L.	Leaves	mg/kg fw	720.2±15.8	4264.2±39.6	2160.8±296.9	395.5±11.6	-	-	-	-	[68]
Chondrilla juncea L.	Leaves	mg/100 g	29.2 (10.6–58.0)	754 (433–1221)	301 (22–472)	40.8 (2.7–73.5)	0.43 (0.12–0.90)	3.97 (1.47–6.57)	0.97 (0.57–1.45)	1.63 (0.53–3.81)	[25]
Cynara cardunculus L.	Flowers	mg/100 g	94	370	44	60	-	1.28	0.256	0.49	[34]
Diplotaxis tenuifolia (L.) DC	Leaves	mg/kg fw	802.3±19.4	5391.4±82.5	2551.0±40.1	541.0±10.8	-	-	-	-	[68]
Foeniculum vulgare Mill.	Leaves	mg/100 g	81.10	618.90	341.30	54.40	0.00	1.30	0.00	0.30	[35]
Malva sylvestris L.	Leaves	mg/100 g	117.4±10.3	757.4 ±78.8	179.70 ± 14.13	715.80 ± 12.1	0,33 ± 0.02	5,82 ± 0.47	0,76 ± 0.04	1,98 ± 0.13	[66]
Montia fontana L.	Leaves	mg/100 g	74.73 ± 13.57	356.50 ± 51.57	31.43 ± 5.38	31.75 ± 3.75	0.05 ± 0.01	1.30 ± 0.26	1.07 ± 0.27	0.38 ± 0.07	[38]
Papaver rhoeas L.	Leaves	mg/100 g	0.00	594 (396-704)	194 (154-233)	140 (120-173)	0.00	1.08 (0.16-2-34)	0.49 (0.39-0.59)	0.28 (0.21-0.33)	[35]
Portulaca oleracea L.	Aerial parts	mg/100 g	21 (7-42)	540 (298-705)	160 (51-234)	143 (56-276)	0.27 (0.16-0.39)	2.16 (0.16-6.82)	0.50 (0.29-0.63)	0.46 (0.21-0.88)	[57]
Scolymus hispanicus L.	Leaves	mg/100 g	39 (11-65)	1040 (559-1773)	235 (124-410)	94 (18-210)	0.09 (0.05-0.13)	2.36 (1.39-3.11)	0.37 (0.16-0.57)	0.50 (0.34-0.92)	[25]
Silybum marianum (L.) Gaertn.	Leaves	mg/100 g	81 (25-128)	718 (432-1300)	132 (42-171)	17 (10-23)	0.08 (0.01-0.17)	0.50 (0.47-0.55)	0.10 (0.03-0.21)	0.26 (0.21-0.35)	[25]
Sonchus oleraceus L.	Leaves	mg/100 g	73.8	319.3	126.6	24.9	0	0.6	0	0.5	[35]
Taraxacum obovatum (Willd.) DC.	Leaves	mg/100 g	34.9 (5.1–61.7)	566 (375–685)	117 (16–269)	18.2 (2.3–34.6)	0.15 (0.08–0.22)	3.57 (2.58–4.18)	0.33 (0.15–0.53)	0.50 (0.22–0.90)	[25]

[¥]Mean value (minimum-maximum)

61. Petropoulos, S.A.; Fernandes, Â.; Tzortzakis, N.; Sokovic, M.; Ciric, A.; Barros, L.; Ferreira, I.C.F.R. Bioactive compounds content and antimicrobial activities of wild edible Asteraceae species of the Mediterranean flora under commercial cultivation conditions. *Food Research International* **2019**, *119*, 859-868, doi:10.1016/j.foodres.2018.10.069.

57. Petropoulos, S.A.; Karkanis, A.; Fernandes, Â.; Barros, L.; Ferreira, I.C.F.R.; Ntatsi, G.; Petrotos, K.; Lykas, C.; Khah, E. Chemical Composition and Yield of Six Genotypes of Common Purslane (*Portulaca oleracea* L.): An Alternative Source of Omega-3 Fatty Acids. *Plant Foods for Human Nutrition* **2015**, *70*, 420-426, doi:10.1007/s11130-015-0511-8.

42. Iyda, J.H.; Fernandes, Â.; Ferreira, F.D.; Alves, M.J.; Pires, T.; Barros, L.; Amaral, J.; Ferreira, I. Chemical composition and bioactive properties of the wild edible plant Raphanus raphanistrum L. *Food Research International* **2019**, *121*, 714-722, doi:10.1016/j.foodres.2018.12.046.

46. Harumi Iyda, J.; Fernandes, Â.; Calhelha, R.C.; Alves, M.J.; Ferreira, F.D.; Barros, L.; Amaral, J.S.; Ferreira, I.C.F.R. Nutritional composition and bioactivity of Umbilicus rupestris (Salisb.) Dandy: An underexploited edible wild plant. *Food Chemistry* **2019**, *295*, 341-349, doi:10.1016/j.foodchem.2019.05.139.

67. García-Herrera, P.; Morales, P.; Fernández-Ruiz, V.; Sánchez-Mata, M.C.; Cámara, M.; Carvalho, A.M.; Ferreira, I.C.F.R.; Pardo-de-Santayana, M.; Molina, M.; Tardio, J. Nutrients, phytochemicals and antioxidant activity in wild populations of Allium ampeloprasum L., a valuable underutilized vegetable. *Food Research International* **2014**, *62*, 272-279, doi:10.1016/j.foodres.2014.03.004.

66. Guerrero, J.L.G.; Madrid, P.C.; Isasa, M.E.T. Mineral elements determination in wild edible plants. *Ecology of Food and Nutrition* **1999**, *38*, 209-222, doi:10.1080/03670244.1999.9991578.

68. Disciglio, G.; Tarantino, A.; Frabboni, L.; Gagliardi, A.; Giuliani, M.M.; Tarantino, E.; Gatta, G. Qualitative characterisation of cultivated and wild edible plants: Mineral elements, phenols content and antioxidant capacity. *Italian Journal of Agronomy* **2017**, *12*, doi:10.4081/ija.2017.1036.

25. García-Herrera, P.; Sánchez-Mata, M.C.; Cámara, M.; Fernández-Ruiz, V.; Díez-Marqués, C.; Molina, M.; Tardío, J. Nutrient composition of six wild edible Mediterranean Asteraceae plants of dietary interest. *Journal of Food Composition and Analysis* **2014**, *34*, 163-170, doi:10.1016/j.jfca.2014.02.009.

34. Gostin, A.-I.; Waisundara, V.Y. Edible flowers as functional food: A review on artichoke (Cynara cardunculus L.). Trends in Food Science & Technology 2019, 86, 381-391, doi:10.1016/j.tifs.2019.02.015.

35. Trichopoulou, A.; Vasilopoulou, E.; Hollman, P.; Chamalides, C.; Foufa, E.; Kaloudis, T.; Kromhout, D.; Miskaki, P.; Petrochilou, I.; Poulima, E., et al. Nutritional composition and flavonoid content of edible wild greens and green pies: a potential rich source of antioxidant nutrients in the Mediterranean diet. Food Chemistry 2000, 70, 319-323, doi:10.1016/S0308-8146(00)00091-1.

38. Tardío, J.; Molina, M.; Aceituno-Mata, L.; Pardo-de-Santayana, M.; Morales, R.; Fernández-Ruiz, V.; Morales, P.; García, P.; Cámara, M.; Sánchez-Mata, M.C. Montia fontana L. (Portulacaceae), an interesting wild vegetable traditionally consumed in the Iberian Peninsula. Genetic Resources and Crop Evolution 2011, 58, 1105, doi:10.1007/s10722-011-9749-7.
